# Tunable Metal–Organic Frameworks for Heat Transformation Applications

**DOI:** 10.3390/nano8090661

**Published:** 2018-08-26

**Authors:** Somboon Chaemchuen, Xuan Xiao, Nikom Klomkliang, Mekhman S. Yusubov, Francis Verpoort

**Affiliations:** 1 National Research Tomsk Polytechnic University, Lenin Avenue 30, 634050 Tomsk, Russia; sama_che@hotmail.com (S.C.); yusubov@mail.ru (M.S.Y.); 2 Laboratory of Organometallics, Catalysis and Ordered Materials, State Key Laboratory of Advanced Technology for Materials Synthesis and Processing; Center for Chemical and Material Engineering, Wuhan University of Technology, Wuhan 430070, China; 265929@whut.edu.cn; 3 School of Chemical Engineering, Suranaree University of Technology, Nakhon Ratchasima 30000, Thailand; nikom.klo@sut.ac.th; 4 Center for Environmental and Energy Research (CEER), Ghent University, Global Campus Songdo, 119 Songdomunhwa-Ro, Yeonsu-Gu 219220, Incheon, Korea

**Keywords:** metal–organic frameworks, heat transformation, low-temperature heat, adsorbent, water adsorption

## Abstract

Metal–Organic Frameworks (MOFs) are a subclass of porous materials that have unique properties, such as varieties of structures from different metals and organic linkers and tunable porosity from a structure or framework design. Moreover, modification/functionalization of the material structure could optimize the material properties and demonstrate high potential for a selected application. MOF materials exhibit exceptional properties that make these materials widely applicable in energy storage and heat transformation applications. This review aims to give a broad overview of MOFs and their development as adsorbent materials with potential for heat transformation applications. We have briefly overviewed current explorations, developments, and the potential of metal–organic frameworks (MOFs), especially the tuning of the porosity and the hydrophobic/hydrophilic design required for this specific application. These materials applied as adsorbents are promising in thermal-driven adsorption for heat transformation using water as a working fluid and related applications.

## 1. Introduction

Energy demand is steadily growing and has become a worldwide issue, especially due to the continuous rise in energy consumption related to the increase in the world’s population. Moreover, energy resources and the exploration of new resources are limited and opposite to worldwide energy demand. Including environmental concerns, the development undertaken towards the exploration of environmentally friendly renewable energy is still challenging. Furthermore, nearly one-third of the world’s energy is consumed for household usage for heat transformation (heating and cooling) [1]. The demand for an air-conditioned environment has rapidly grown due to higher living standards, increased climate temperatures, as well as the modern design of architecture. Also, there is an increasing concern over ozone depletion related to the greenhouse effect resulting from applied compounds, e.g., Chlorofluorocarbons (CFCs) and hydrofluorocarbons (HFCs) in household systems for heat transformation. In the meantime, the development and deployment of new heat transformation system technologies that are energy-saving and environmentally friendly offer great potential to sustain and address the ever-increasing worldwide energy demand. Thermally driven adsorption chillers and adsorption heat pumps in heat transformation systems based on the reversible adsorption/desorption of the working fluid that apply porous adsorbent materials are very promising. The more efficient use of low-temperature heat and an effective adsorption approach, as well as effective climate protection through the reduction of the environmental impact of conventional heating and cooling devices, is encouraging. The main principle of the process is based on sequential adsorption/desorption procedures [2]. Large advances in the enhancement of heat transformation efficiency can be accomplished with advances in the design of new adsorbent materials. Therefore, current progress in this field is essentially related to the development of innovative materials which are properly adapted to this target. Currently, a broad exploration has been devoted to the design, synthesis, and development of new adsorbent materials with an ability for water adsorption that exceeds that of existing commercial materials (e.g., silicas or zeolites) and in the meantime possess relatively gentler regeneration conditions.

Micro- and mesoporous materials are significantly developed and exhibit potential as adsorbent materials for heat transformation systems [3]. Nonetheless, most of the available adsorbents used for heat transformation systems have originally been developed for gas separation and catalysis [4,5]. Silica gels and silica-aluminophosphates are the most reported adsorbent materials for heat transformation systems due to the advantageous high adsorption capacity of the working fluid, which is mostly water. However, the working fluid (water) is mostly uptaken at too high a relative pressure, which is the main problem when using the current adsorbent materials, such as silica. The resulting loading difference (in grams of adsorbed working fluid/kilogram of adsorbent) of the adsorbent over a given cycle, or the cycle efficiency, is small compared to the total adsorption capacity [6]. Therefore, there is an ongoing search for new materials with higher water uptake capabilities, which are required to improve the system performance of a heat transformation system. In this respect, new materials, such as metal–organic frameworks (MOFs) generated from metal ions/clusters bridging with organic linkers, have great prospects as adsorbents to replace the currently applied adsorbents [7]. During recent years, these materials have had booming development and burgeoning potential applications, such as gas adsorption and storage [8], separation [9], catalysis [10], sensing, drug delivery [11,12], biomedicine [13], and so on [14,15,16,17]. As their structures can be constructed with a diversity of metal clusters/ions and organic components and generate porous materials with a high surface area, MOFs possess more advantages to tune their properties via structure design. Moreover, the modification of MOFs via functionalization or post-synthetic modification could offer attractive routes to enhance their properties for heat transformation and related applications.

## 2. Heat Transformation Systems

**Figure 1:**
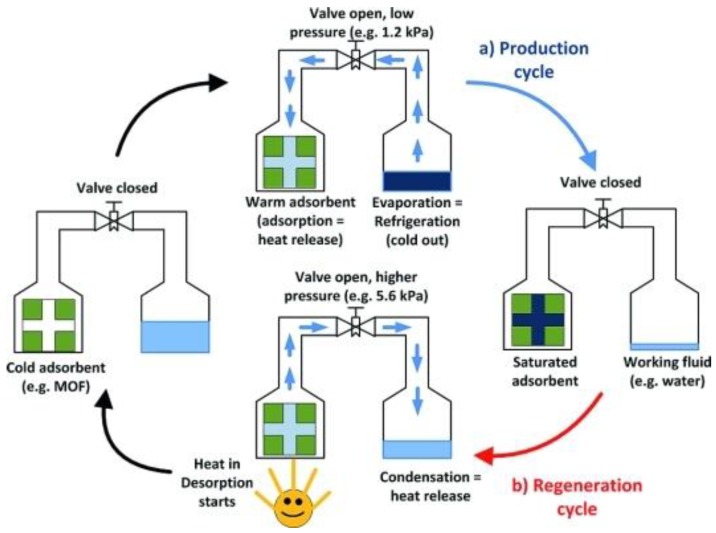
Illustration of the basic principle for adsorption chillers or heat pumps. Reproduced with permission from [18]. Wiley, 2011. MOF, metal–organic framework.

The thermodynamic principle of a heat transformation system using a solid adsorbent with the work produced by the heat engine driving the heat pump cycle is demonstrated in Figure 1. Basically, an ideal cycle in a heat transformation application consists of two processes: vapor (working fluid) adsorption on highly porous solid materials (adsorbent) followed by the regeneration or desorption cycle. In the adsorption cycle, the working fluid is vaporized at a low temperature, thereby producing the beneficial cooling. When the vapor of the working fluid is adsorbed in the pores of the adsorbent, it releases the adsorption heat at a medium temperature level. This adsorption heat can be used as useful heat in the heat pump case or withdrawn from the environment in case of cooling. As adsorption is an exothermic process, the adsorbent extracts heat from the evaporator and produces a cooling effect. Later, in the regeneration process or desorption cycle, a heating source having a high temperature (e.g., a solar thermal collector or gas burner) is used to desorb the vapor from the adsorbent material. The released adsorbed vapor condenses at a medium temperature level (ambient temperature) with the heat of condensation liberated into the environment. As desorption is an endothermic process, it helps to release the refrigerant (water vapor) from the hot bed. After that, the hot refrigerant will be cooled in a condenser to feed the evaporator with the refrigerant liquid and the cooling process is maintained in a continuous manner.

In a standard process, the sorption system exchanges the fluid vapor (evaporator/condenser) between the liquid phase and the adsorbent material. The working fluid is important in the process since it has a specific evaporation enthalpy. Preferably, water is applied as a working fluid; however, other fluids, such as ethanol, methanol, and acetone, also can be applied for specific purposes. Nevertheless, adsorbent materials are most important regarding the process’s efficiency to adsorb the vapor of the working fluid and highly porous solid materials, such as MOFs, exhibit this property.

**Figure 2:**
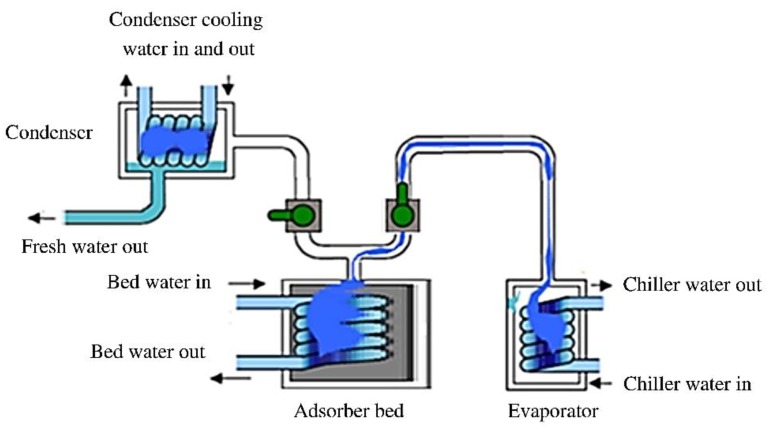
Schematic diagram for one-bed adsorption desalination. Reproduced with permission from [20]. Elsevier, 2017.

In another direction of adsorbent materials applied for heat transformation, which is based on vapor adsorption of the working fluid (water), the heat transformation system is operated in a closed system and the working fluid exchange phase (vapor/liquid) via heat transformation from the environment has a cooling or heating application. However, using adsorbent materials with great ability to adsorb fluid vapor (water) from an open environment followed by desorption using a high temperature from natural heat to condense the fluid (desorption) has been another route of application of these materials. Desalination is generally defined as the heat transformation process by which potable water is produced from seawater or brackish water with a high dissolved suspended solid content (35,000 ppm). Desalination processes can be divided into two major groups: the first group is a heat-driven process (distillation), such as multi-stage flashing (MSF), multiple effect distillation (MED), and solar distillation. The second group employs electric-power-driven processes that include freezing, mechanical vapor compression, electrodialysis, and reversed osmosis. All the desalination systems suffer from two drawbacks; namely, they are highly energy intensive and/or prone to fouling and corrosion of the evaporator or separation unit of the sea water [19]. Recently, a new process of adsorption desalination using an adsorbent has been created with several benefits, including being environmentally friendly and driven by low-grade heat sources and having a low capital cost, a low evaporation temperature, and hence a reduced fouling (formation of scales causing damage to the evaporation units) effect. The principle system consists of an evaporator, a condenser, and an adsorption/desorption station. Each station includes a finned tube heat exchanger containing a solid adsorbent material between the fins. Basically, seawater is firstly vaporized producing water, which is then adsorbed by the adsorbent material. Then, during the desorbing step by heating the station, the water vapor is desorbed and then condensed in the condenser producing high-grade distilled water (Figure 2) [20]. Different adsorbent materials, such as silica gel and zeolite, have been reported for the desalination application [21,22,23].

## 3. Metal–Organic Frameworks as Adsorbent Materials

It is clear that adsorbent materials are key in the development of heat transformation technologies. In this respect, the discovery of new materials applicable to an adsorption-desorption working fluid is still fundamental research of which the development of this technology is ongoing [24,25,26,27]. Porous coordination polymers (PCPs), also well-known as metal–organic frameworks (MOFs), have demonstrated excellent properties as adsorbents and are explored for their heat transformation applications. Due to their huge surface (having micro- to mesopore-pores) and chemical and physicochemical variability, tunable composition/properties can be designed. Moreover, MOFs consist of both hydrophilic and hydrophobic moieties in the same structure, which possess some unique adsorption properties. Compared with a vast number of natural and synthetic adsorbents, MOF materials have high potential for heat transformation applications because of their great ability to adsorb guest molecules, including water (working fluid), and thermal stability [24,28]. Additionally, MOFs possessing hydrophilic properties have an advantage over silica gel as they exhibit a non-limited water uptake at high relative pressure values reaching their maximum capacity. Initially, the demonstration of MOFs as adsorbent materials was carried out by investigating their capability in solid–gas adsorption applications for energy transformations. Energy storage and heat transformation (cooling/heating) using MOFs materials proved to have great potential applications. The investigated adsorbent materials were characterized via water adsorption-desorption properties since water is mostly used as a working fluid. In addition, MOFs have been considered for water adsorption measurements in order to investigate the structural properties and adsorption performances. In the water adsorption process using MOFs, the metal clusters firstly coordinate water molecules before the capillary condensation mechanism in the pores of the solid adsorbent (MOFs) can occur [29]. Therefore, the types of metal clusters are used to classify MOF materials for water adsorption with potential for heat/energy transformation applications. Additionally, for some frameworks, a geometric flexibility and reversible change in the structure related to the guest adsorption was observed. Consequently, water adsorption on MOFs was used to evaluate their performance for heat transformation applications.

Compared to the present adsorbents applied for heat transformation applications, such as zeolites or aluminophosphates, MOFs exhibit a much higher potential for this application due to their composition, pore structure, and topology. Moreover, further improvement via modification or functionalization of the metal clusters/ions and organic linkers is still possible, as well as the optimization of the porous structure of the MOFs, which allows for the tuning of their adsorption properties [30]. This opens attractive prospects for the design of MOFs with predetermined properties adopted for operating conditions particularly suitable to heat transformations [28]. Remarkably, progress in MOF chemistry has advanced and the deployment of several methodologies to synthesize and modify water-stable MOFs has paved the way for water-sorbent candidates with enhanced ability for water uptake and related applications (Table 1) [31,32,33,34,35]. From a qualitative point of view, the adsorption properties of MOFs are obviously quite diverse in terms of water uptake capacity and the associated relative pressure at which the pore filling occurs. However, hydrolytically stable porous materials offering remarkable pore volumes are expected to exhibit large water adsorption capacities. The quest for hydrolytically stable and recyclable MOFs with superior total water uptake remains a focal point of intensive research in MOF chemistry [32,36,37,38].

**Figure 3:**
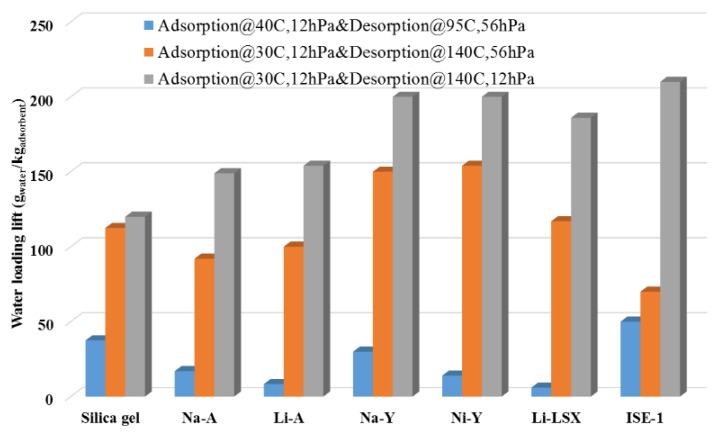
Water loading spread over the cooling cycle for various adsorbents. Reprinted with permission from [54]. American Chemical Society, 2011.

**Figure 4:**
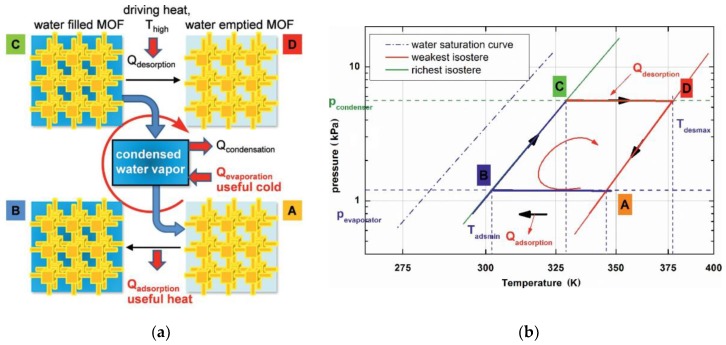
Principle process of the ideal cycle in an adsorption heat-pump or chiller process (**a**) and diagram (**b**). A→B: production or adsorption cycle. C→D: regeneration or desorption cycle Reproduced with permission from [18]. Wiley, 2011; [45]. Wiley, 2011.

The most common energy transformation is in sensible heat storage and latent storage. A higher thermal storage efficiency via increasing the thermal storage capacity (sensible heat capacity) is required to lower the volume of a sensible thermal storage system. A working fluid based on water applied to heat storage and transformation has the highest mass-based evaporation enthalpy (2440 kJ/kg at 298 K) and an adsorption enthalpy comparable to all known fluids [54]. To investigate a suitable candidate, adsorbents were measured via adsorption equilibrium data points corresponding to common cycle conditions (at the end of the adsorption and desorption half-cycle). The results demonstrated the outstanding performance of MOFs compared to silica gel and several zeolites (Figure 3) [54]. Figure 3 displays that at a higher desorption temperature of 413 K against a lower condensation pressure of 12 hPa (total height of the bars), the loading spread achieved with the new material MOF (ISE-1) amounts to ∼210 g/kg and is larger compared with all five zeolites and the reference silica gel. To date, several MOFs, such as MIL-101 [55], MIL-100, mp-AF or Basolite™ A520 [56], NH_2_-MIL-125 [57], and UiO-66 [48], have been explored to adsorb working fluids, such as water and methanol, for a heat transformation system (Figure 4). This review aims to provide promising MOF candidates with a special focus on sorption processes based on the evaluation of the water uptake characteristics.

### 3.1. Thermodynamic Boundaries of MOF Adsorbents

The adsorption equilibria and the applied adsorption material are used to illustrate the heat transformation cycles. The first state of merit is the achievable working fluid (water) loading on adsorbent materials; more precisely, the working fluid exchange between the production cycle (adsorption) and the regeneration cycle (desorption). Using the van ’t Hoff diagram in Figure 4, the exchange as the difference between the richest and the weakest isosteric of the cycle based on a MOF adsorbent can be described [45]. The minimum adsorption (point B) and the maximum desorption temperature (point D) by the evaporator and condenser pressure were used to determine the process, while in the thermodynamic context, the isosteric term occurs at a constant water loading without adsorption or desorption. In the case of the cooling application (A→B), the water is evaporated and heat is adsorbed from the environment producing useful chilling (cooling case). The cooling enthalpy produced in one cycle can be calculated simply as the evaporation enthalpy multiplied by the working fluid exchange. In the meantime, the heat of adsorption is released to the environment (in the case of a heat-pump application), which is the useful heat. The cycle is defined by the highest desorption temperature (driving temperature, point D), the minimum adsorption temperature (point B), and the condenser and evaporator pressure. During isosteric heating (B→C), the pressure level increases to the condensation pressure level; thereby, the selected pressure level is geared to the possible applications. Then, the saturated adsorption on the adsorbent material is regenerated (C→D), and heating from a high-temperature source (e.g., a solar-thermal collector, waste heat) is used to desorb the working fluid from the adsorbent material. Finally, the isosteric cooling (D→A) closes the cycle. In an ideal cycle, adsorption and desorption are supposed to be an isobaric process. Consequently, a systematic material determination for this kind of use is realized by quantifying the two isobars corresponding to the condenser and evaporator pressure.

Suitable adsorbent materials for these applications are often wrongly understood in such a way that they should provide a large adsorption capacity of the working fluid, which obviously would indicate a favorable strong interaction between the adsorbent and the adsorbate. Consequently, a too-high temperature is required for the desorption process, which may not be available from natural heat sources. Therefore, suitable adsorbent materials depend on the cycle boundary conditions, which basically are discussed in the diagram given in Figure 4b. A promising adsorbent material should exhibit a large different working fluid adsorption (∆*W*, *g*_water_/*g*_adsorbent_) between the richest (B→C) and the weakest (D→A) isostere of the cycle, as ∆*W* = *W*_max_ – *W*_min_. The adsorption *W*_max_ (richest isostere B→C) is characterized by two temperature/pressure couples (*T*_adsmin_:*T*_B_, *P*_evaporator_:*P*_E_ and *T*_point c_:*T*_C_, *P*_condenser_: *P*_C_), while *W*_min_ (weakest isostere D→A) is characterized by *T*_desmax_: *T_D_*, *P*_condenser_: *P*_C_ and *T*_point A_: *T*_A_, *P*_evaporator_: *P*_E_. This can be simplified in the adsorption potential ∆*F* = −RTIn(*P/P_0_*), which obeys the Polanyi potential theory, where P_0_ is the saturation vapor pressure at temperature *T* [58,59]. In this case, the richest and weakest isosteres are unambiguously defined by the potentials ∆*Fr* = −RT_B_ln[*P*_E_/*P*_0_@*T*_B_] = RT_C_ln[*P_C_*/*P*_0_@*T*_C_] and ∆*F*w = −R*T*_D_ln[*P*_C_/*P*_0_@*T*_D_] = R*T*_A_ln[*P*_E_/*P*_0_@*T*_A_], and the difference ∆*W* = *W*(∆*F*r) – *W*(∆*F*w) should be maximized. If the uptake is invariant with respect to the relative pressure *P/P_0_*, the increment ∆*W* = *W*[*P*_E_/*P_0_*@*T*_D_] – *W*[*P*_E_/*P*_0_@*T*_A_] is the optimization parameter. From a practical point of view or for industrial applications, optimization depends on the application condition or operation parameter, such as type of working fluid, cooling or heating system, temperature requirement, and working pressure; however, this is out of the scope of this review.

### 3.2. Copper-Metal-Based MOFs

**Figure 5:**
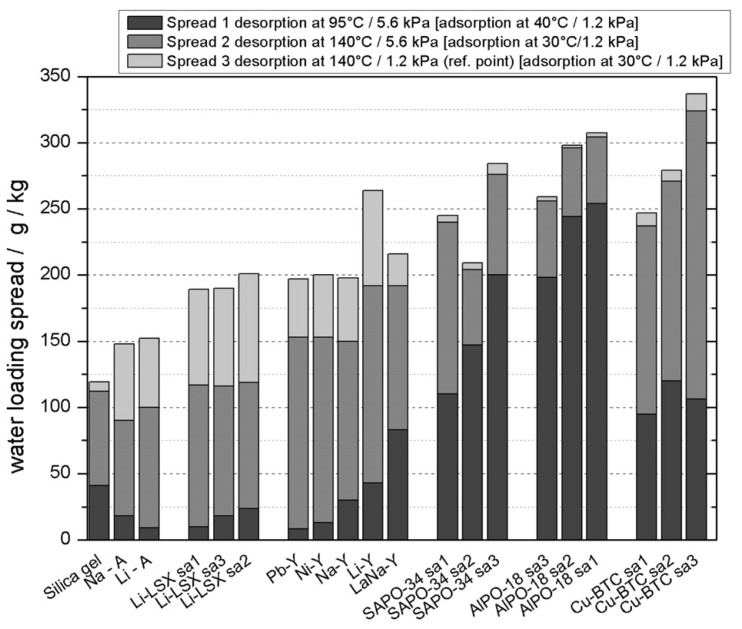
Comparison of water loading spread over three cycle conditions for different materials. Reproduced with permission from [24]. Elsevier, 2010.

HKUST-1 or Cu_3_(BTC)_2_ is the most frequently investigated MOF for a variety of applications. Chui et al. were the first to produce a copper (Cu^2+^) metal clusters bridging with a benzene-1,3,5-tricarboxylate linker (BTC) [60]. HKUST-1 contains an intersecting three-dimensional (3-D) system of large square-shaped pores of 9 × 9 Å and other smaller pores having a diameter of 6 Å. In the framework of HKUST-1, Cu (II) ions form dimmers, where each copper atom is coordinated by four oxygens from the BTC linkers and water molecules. Moreover, HKUST-1 was the first available MOF having outstanding properties among the commercially available MOFs as shown by its interesting properties, such as its gas storage, gas separation, and catalytic performance [61,62]. HKUST-1 demonstrated also an impressive performance in water adsorption of about 0.095–0.120 *g*_water_/*g*_adsorbent_ at 313 K/1.2 kPa and 0.324 *g*_water_/*g*_adsorbent_ at 303K/1.2kPa, which is much higher than that of the currently used adsorbents, such as silica gel (Figure 5a). The presence of water molecules in the first coordination sphere of Cu ions has been suggested as a possible reason to obtain a coordinative vacancy on Cu(II) species. The second step in the adsorption isotherm indicates that the water molecules are filling the pores of the solid adsorbent (first the large pores followed by the small pores) due to a more hydrophobic character, since no available metal sites are accessible. Additionally, the hydrophobic character of the benzene linker enhances the hydrophobicity of the small pores. They are less hydrophilic as the pore’s interior is constituted by four benzene rings [63]. A saturation is attained at about *P/P*_0_ = 0.4. An additional increase of *P/P*_0_ = 0.9–1.0 may result from the condensation of H_2_O in the inter-particulate volume. The desorption exhibits a slight hysteresis due to strong hydrogen bonds formed between the water molecules [24]. Note that not all water molecules are desorbed in the lower pressure region. This phenomenon is probably the result of the chemisorption of water molecules on the free copper coordination sites. It is noteworthy that the pre-treatment and different preparation procedures (solvent used, crystal size, etc.) also affect the performance of adsorption. Unfortunately, HKUST-1 was found to be significantly unstable during a recycling experiment.

MOF materials having a pillared-layer structure were synthesized by Kitagawa [64] using Cu^2+^, Na_2_pzdc (pzdc = pyrazine-2,3-dicarboxylate), and a series of pillar ligands of pyridine derivatives. The structure of Cu_2_(pzdc)_2_(dpyg)]*_n_* (pzdc = pyrazine-2,3-dicarboxylate; dpyg = 1,2-di(4-pyridyl)glycol) with a flexible and functional pillar was examined for the adsorption of CH_4_, MeOH, and H_2_O [64]. The MeOH adsorption isotherm displays a sudden rising at relative pressure (*P/P_0_*) 0.23 and reaches a saturated level (ca. 0.2 *g*_MeOH_/*g*_adsorbent_) at point *P/P_0_* = 0.5. For the reversed cycle, the desorption isotherm reveals non-correspondence with the adsorption isotherm (hysteretic loop); instead, a sudden drop at *P/P_0_* = 0.1 is obtained. Moreover, this hysteretic shape between the adsorption and desorption isotherms was reproducible numerous times. The sharp adsorption jump/desorption fall within the hysteresis loop indicates a crystal phase change or transformation occurrence in the MOF framework, which should permit the inclusion of guest molecules. This effect is mainly associated with the hydrogen bond interaction between the MeOH molecules and the OH groups of the dpyg ligands; the attractive force should be strong enough to transform the channel structure to allow for the incorporation of the guest molecules. Therefore, the pillared-layer motif design for the rational synthesis of MOFs based on the response of the flexible and dynamic framework of specific guest molecules could enhance porous materials with a higher adsorption ability.

### 3.3. Chromium (Cr)-Based MOFs

**Figure 6:**
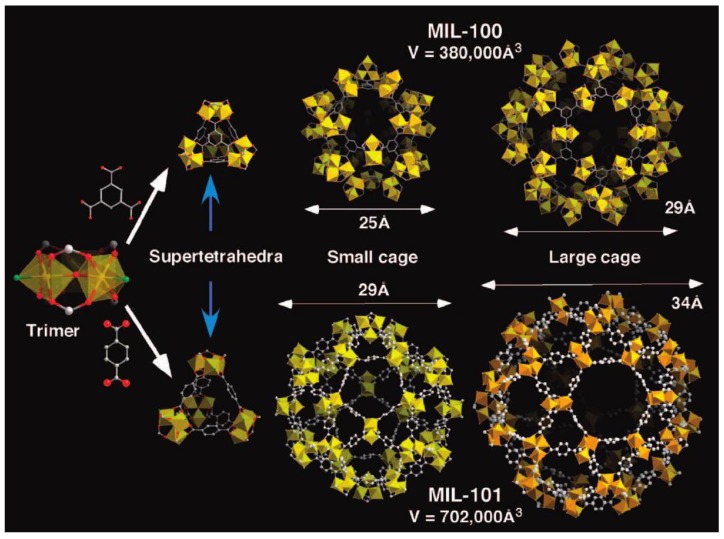
Structures of MIL-100 and MIL-101. Reproduced with permission from [67]. American Chemical Society, 2008.

**Figure 7:**
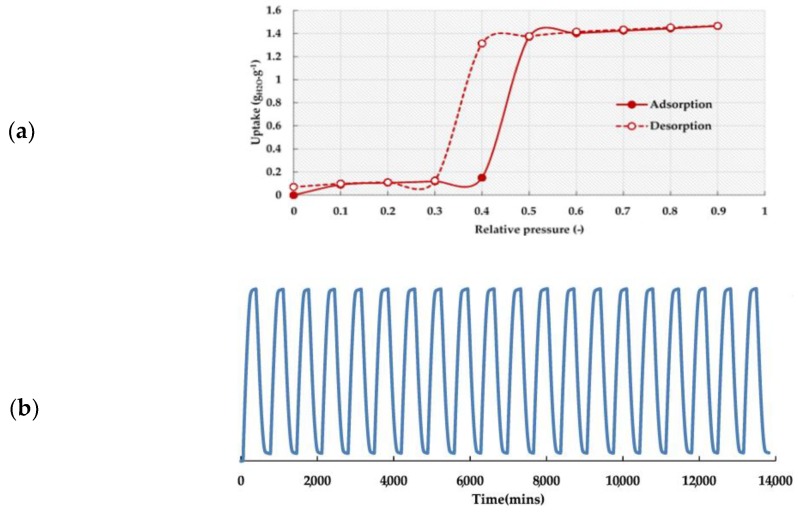
(**a**) Water adsorption isotherms of MIL-101 at 298 K and (**b**) Adsorption/desorption performance of MIL-101(Cr) at 298 K. Reproduced with permission from [69]. Elsevier, 2017.

MILs (acronym for Matérial Institute Lavoisier) have extensively been investigated in MOF categories and are constructed from various metal clusters (Cr, Fe, Al, Ti, etc.) with an organic linker, such as terephthalic acid. Férey et al. were the first to synthesize and report the chromium-terephthalate-based solid (MIL-101) [65]. The structure is built up from a Cr octahedral with rigid carboxylate ligands (terephthalate or trimesate) generating microporous super-tetrahedral units (Figure 6). The connection of these motifs provides an augmented version of the 3D MTN-type (Framework type MTN or ZSM-39) zeolite topology [66]. The resulting cubic cell volumes are huge (∼380,000 and 702,000 Å), with two types of porous cages limited by pentagonal faces for the smaller cage and by pentagonal and hexagonal faces for the larger cage. After removal of the guest molecules, the free internal diameters are close to 2.9 and 3.4 nm. The cages are accessible through microporous windows of 1.2 and 1.6 nm [67]. MIL-101 showed exceptional properties, such as a high pore volume (2 cm^3^/g), a large surface area (4500 m^2^/g), being water stable, and a high thermal stability [68]. Water adsorption/desorption was measured at 298 K and exhibited the adsorption *type IV* isotherm (Figure 7a). The adsorption mechanism revealed that the water vapor adsorption is related to the unsaturated metal centers (UMCs) at low relative pressure (*P/P_0_* <0.4). Nevertheless, the hydrophobic properties of the ligand of the MOF prohibited further water uptake. At a higher relative pressure (*P/P_0_* = 0.4–0.5) a steep increase in water uptake was found due to the involvement of capillary condensation in the mesopores. At higher relative pressure (*P/P_0_* >0.5) a stable water uptake was observed as a result of the full filling of the pores with water. Since most adsorption occurs in the large pores of the material, a hysteresis loop between the adsorption and desorption branches of the adsorption isotherms is obtained. Furthermore, the material demonstrated a high hydrothermal stability since over 20 adsorption/desorption cycles could be performed successfully (Figure 7b).

Extension of the design of Cr-based MOFs to obtain a larger water uptake while retaining their chemical and thermal stability was investigated via enlargement of the porosity to obtain ultra-high surface areas. The Cr-cluster was bridged using an expanded linker of 3,3’’,5,5’’-tetrakis(4-carboxyphenyl)-p-terphenyl (H_4_TCPT) and given the abbreviation Cr-soc-MOF as reported by Edouaddi [38,70]. The Cr-soc-MOF material exhibited high porosity, 4549 m^2^/g of surface area, and a large pore volume of 2.1 cm^2^/g after traditional activation (a heating and vacuum process). The extremely high porosity of Cr-soc-MOF was beneficial to increase the water adsorption ability. A water vapor uptake of 1.95 *g*_water_/*g*_adsorbent_ was observed for this adsorbent material under a relative humidity of 70%. Moreover, the exceptional thermal and chemical stability was proven via more than 100 adsorption/desorption cycles, after which the framework structure was still unchanged. The authors claimed that the Cr-soc-MOF exhibited an extremely high water uptake and stability compared with any other reported Cr-based MOF.

### 3.4. Nickel (Ni)-Based MOFs

**Figure 8:**
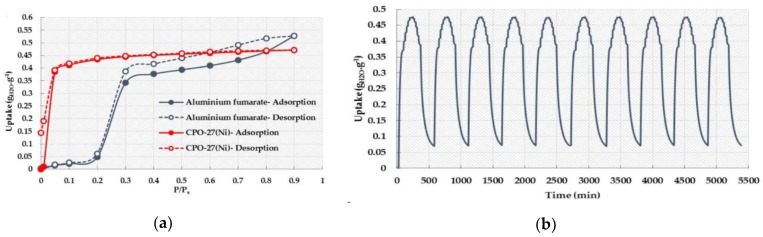
(**a**) Water adsorption isotherms of CPO-27(Ni) at 298 K and (**b**) Adsorption/desorption cycling experiments for CPO-27(Ni). Reproduced with permission from [72]. Elsevier, 2016.

MOF-74(Ni), also known as CPO-21-Ni (CPO: Coordination Polymer of Oslo), is a hydrothermally stable metal–organic framework [71]. The crystal structure of CPO-27 is based on a honeycomb motif with pores of 12 Å diameter and helical chains of edge-condensed metal-oxygen-coordinated octahedra located at the intersections of the honeycomb. Water adsorption was performed on the material at 298 K and the obtained result showed that the material exhibits a maximum uptake of 0.47 g_water_/g_adsorbent_ at a relative pressure of 0.9 (Figure 8). Noteworthy is that it has been reported that the water adsorption at low-pressure values is related to unsaturated metal centers (UMCs) [72]. UMCs were formed after the removal of guest molecules on the metal atoms and these UMCs can easily attract water molecules via these extra binding sites. Moreover, the presence of a hydroxyl group on the organic linker, a hydrophilic advantage, is also involved in this phenomenon. On the other hand, strong interaction at the binding sites with the guest molecules requires strong conditions for the regeneration process. In order to evaluate the performance stability, adsorption/desorption experiments were conducted using CPO-27(Ni), which demonstrated excellent performance stability during 10 cycles with a negligible decrease (0.35%).

### 3.5. Aluminum (Al)-Based MOFs

**Figure 9:**
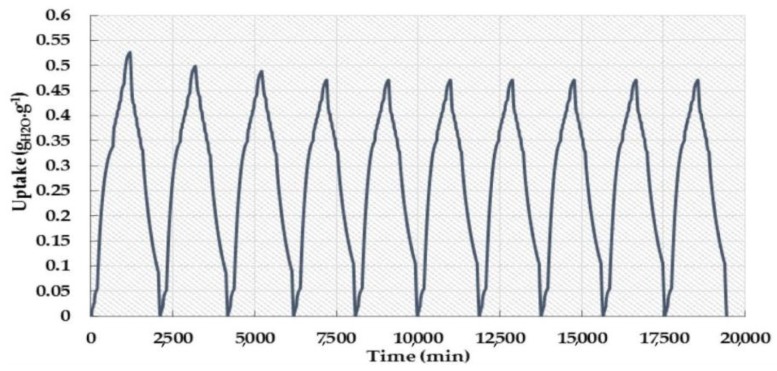
The adsorption/desorption experiments for aluminum fumarate. Reproduced with permission from [72]. Elsevier, 2016.

Aluminum bridged with fumaric acid (FA) as an organic linker formed the MIL-53(Al)-FA structure having the formula Al(OH)(fum)∙*x*H_2_O (*x* = 3.5; fum = fumarate) and exhibiting an structure isoreticular to the well-known material MIL-53(Al)-BDC or Al(OH)(BDC)∙H_2_O (BDC=1,4-benzenedicarboxylate). The framework is constructed from chains of corner-sharing metal octahedra linked together by fumarate to form lozenge-shaped one-dimensional (1D) pores having circa 5.7 × 6.0 Å free dimensions. As expected, these sizes are smaller than those observed for the parent terephthalate open forms (7.3 × 7.7 Å for MIL-53(Al)-BDC (as-synthesized) and 8.5 × 8.5 Å MIL-53(Al)-BDC (at high temperature), which is consistent with the shorter length of the fumaric acid compared to the terephthalic acid [73,74]. The water uptake in this material reached 0.53 *g*_water_/*g*_adsorbent_ at *P/P_0_* = 0.9 (Figure 8a). The adsorption isotherm exhibited a type IV isotherm with hysteresis, indicating a narrow distribution of uniform pores. The limited water adsorption at low relative pressure (*P/P_0_* <0.2) is related to the hydrophobic properties of the organic linker. The steep increase in the water uptake at *P/P_0_* = 0.2–0.3 is followed by a continuous increase till the maximum adsorption is reached. The adsorption mechanism for the uniform accumulation of water molecules in the inner pores of the material has been reported as well [71]. It followed that a higher relative pressure is required to induce pore filling with water vapor [29]. Water adsorption/desorption cycles were performed and showed a steep decrease during the first four cycles followed by a stabilization of the adsorption/desorption up to 10 cycles (Figure 9) [72].

CAU-10-H is one of the commercial MOFs that contains isophthalic acid as an organic linker and cis-connected AlO_6_-polyhedra forming helical chains. The potential of CAU-10-H is further strengthened by the fact that this material demonstrates a higher volumetric adsorption capacity and thermodynamic efficiency for water [75]. The highly pure crystalline phase is perfectly stable towards water and has not shown any sign of degradation over 700 repeated adsorption/desorption cycles, a feature not commonly encountered for MOFs when exposed to water [76]. The water adsorption isotherm shows the required *S*-shape with a steep rise at *P/P_0_* = 0.2, a maximum uptake of approximately 0.33 *g*_water_/*g*_adsorbent_, and nearly no hysteresis. The material shows a structural transition indicating a flexibility of the material through adsorption and desorption, the so-called breathing effect [76]. Despite or because of this flexibility, CAU-10-H is stable over 10,000 cycles of adsorption/desorption. The unique shape of the isotherms allows for the use of very low desorption temperatures of less than 348 K at condensation temperatures around 303 K [77].

**Figure 10:**
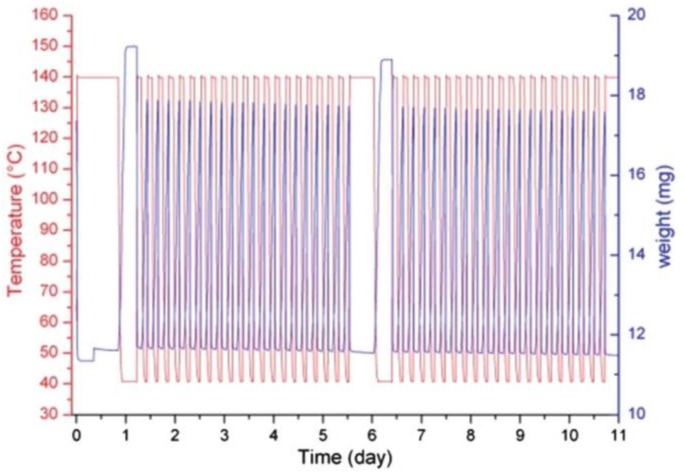
Temperature profile and load signal of the MIL-100(Al) cycling experiment acquired at p*_H2O_*= 5.6 kPa. Reproduced with permission from [43]. Royal Society of Chemistry, 2012.

MIL-100 with the empirical formula 3D-(Al_3_(btc)_2_∙*n*H_2_O) (btc = benzene-1,3,5-tricarboxylate, trimesate) has received plenty of interest in the fields of catalysis, gas separation, and gas storage [78,79,80]. Water adsorption behavior was obtained at small relative pressures (*P/P_0_* <0.25) due to adsorption and cluster formation at the hydrophilic metal sites of the compound. The steep rise at 0.25 < *P/P_0_* < 0.45 has been soundly explained with the consecutive filling of first the 25 Å and then the 29 Å pores. The low water adsorption of MIL-100(Al) (0.5 *g*_water_/*g*_adsorbent_) was explained by the incomplete H_2_O filling of the pores. Additionally, the presence of hydrophobic sites, e.g., small amounts of unremoved H_3_btc, may inhibit the complete wetting of the pore walls [43]. The hydrothermal cycle stability of MIL-100(Al) was quantified with samples exposed to a humidified gas flow (Figure 10). This material was relatively water stable with small sub-sequential losses of water capacities and porosities and no detectable loss of crystallinity (XRD).

### 3.6. Iron (Fe)-Based MOFs

Another porous MOF, MIL-100(Fe), consists of iron (III) tricarboxylates as metal clusters bridging with tricarboxylate linkers constructing supertetrahedral cages. The pore diameter of the supertetrahedra is 6.6 Å, which is slightly smaller than the pore diameter in MIL-101(Cr) [80]. The specific surface area obtained via nitrogen adsorption measurements at 77 K revealed a Langmuir surface area larger than 2800 m^2^/g. The polymodal pore size distribution in MIL-100(Fe) possesses both micropores and mesopores. Additionally, water adsorption isotherm measurements revealed that water molecules were adsorbed in the mesopores at the higher-pressure region, which is typically observed for microporous materials with a more or less polar inner surface, such as HKUST-1. The steep adsorption step at *P/P_0_* = 0.3–0.4 indicates the filling of the mesoporous cages. Similar to HKUST-1, adsorption first appears on the vacant metal sites in the framework. Thereafter, the mesopores are filled consecutively via adsorption, of which first the smaller 25 Å pores are filled and then the larger pores (29 Å pores). The saturation is obtained at *P/P_0_* = 0.5 and there is only a slight additional increase of the adsorbed water volume, which is due to the adsorption of molecules in the inter-particulate voids of the material. The water adsorption capacity of as-synthesized MIL-100(Fe) can reach as high as 0.75 *g*_water_/*g*_adsorbent_ at 298 K [43]. The desorption branch of MIL-100 (Fe) obtained a distinct hysteresis, especially for the large mesopores. This phenomenon is widely known for mesoporous materials, yet is undesirable for the intended application as it considerably decreases the usable part of the loading. Since MIL-100(Fe) is synthesized in water, it should have high water stability. Additionally, this MOF is based on iron, which is much more suitable for industrial applications than other metal-based MOFs containing metals, such as copper, chromium, and cobalt, regarding health and toxicity.

## 4. Modification/Functionalization of MOFs Based on Water Adsorption

One of the advantages of MOF materials compared with other conventional porous material adsorbents is not only the tunable nature with the choice of metal ions and the linker species but also the modification or functionalization ability to tune their properties. Several MOFs have been briefly introduced in the previous section. However, there are still major challenges to overcome to solve the drawbacks of these materials, such as stability, uptake performance, and handling, before they can be used in practical applications. 

### 4.1. Functionalized MOFs Bearing a Ligand Functional Group

The largely available porosity in materials is not the only parameter to exhibit a high ability for water adsorption of a porous adsorbent. The tuning of hydrophobic/hydrophilic properties of organic linkers with the hydrogen-bonding capabilities of functional groups is also a possibility for a functional structural transition of the adsorbent material [52,53,81]. In order to tune the water uptake to lower *P/P_0_* values, the organic linker can be modified with a hydrophilic functional group before assembling the MOF. Several functional groups in the ligand molecules, such as nitro-terephthalate (-NO_2_), amino-terephthalate (-NH_2_), and sulfo-terephthalate (-SO_3_H), have been used to construct MOFs and have been investigated for their water adsorption behavior in comparison to the non-functionalized ligand-containing MOFs. The MIL-101 and MIL-101 derivatives were used to elucidate the effect of ligand functional groups on water adsorption [46]. Different isotherm lines were found among the water adsorption of MIL-101, MIL-101-NO_2_, MIL-101-NH_2_, and MIL-101-SO_3_H. It is worth noting that the isotherm lines of MIL-101-NH_2_ and MIL-101-SO_3_H shifted to lower *P/P_0_* values compared with that of MIL-101, which was attributed to the highly hydrophilic groups on their pore surfaces. In contrast, the isotherm of MIL-101-NO_2_ shows almost the same profile as that of MIL-101 in terms of water uptake pressure, which might be because of the lower hydrophilicity of the NO_2_ group. The introduction of a hydrophilic group, such as -NH_2_ or -SO_3_H, could provide a hydrophilic environment inside the pore, resulting in a stronger interaction between the pores and water molecules. As a result, MIL-101-SO_3_H and MIL-101-NH_2_ start to adsorb water in a lower pressure region than that for the original MIL-101. Similar results were obtained for the study of a 2-amino benzene dicarboxylic acid (NH_2_-BDC) bridging as a linker of cyclic octamer corners or edge-sharing TiO_5_(OH) octahedrons generating Ti_8_O_8_(OH)_4_-(O_2_CC_6_H_5_-CO_2_-NH_2_)_6_ or NH_2_-MIL-125. Kim et al. demonstrated that water adsorption on NH_2_-MIL-125 showed a sharp rise of adsorption at a relative pressure of about (*P/P_0_*) 0.2, making this MOF a promising new candidate for adsorptive air conditioning driven by low-grade heat [82]. The study indicates that the carboxylate and the O-Ti-O groups are primary adsorption centers forming hydrogen bonds with water molecules. However, the highly hydrophilic -NH_2_ groups could be another type of primary adsorption center. To verify the hydrothermal stability of NH_2_-MIL-125, the material was subjected to multiple adsorptions/desorption cycles applying conditions such as *P* = 2.36 kPa, *T*_ads_ = 313 K, and *T*_des_ = 383 K. The experiment revealed that the water uptake displayed a small decrease during the first cycle (from 0.42 to 0.40 *g*_water_/*g*_adsorbent_); thereafter, the uptake turned out to be stable (w = 0.39 *g*_water_/*g*_adsorbent_) across the remaining cycles. The texture characteristics of the studied MIL after 10 cycles (NH_2_-MIL-125-10АС) changed only slightly with the specific surface area *S*_sp_ dropping from 1300 to 1230 m^2^/g and the pore volume *V*_p_ declining from 0.56 to 0.54 cm^3^/g.

The effect of halogen ions (F, Cl) and sulphate (SO_4_) incorporated into the MOF structure on water adsorption was investigated using MIL-100(Cr) [42]. The free counter anion in the MOF pores was replaced with other counter anion using different inorganic acids (F = hydrofluoric acid, Cl = hydrochloric acid, and SO_4_ = sulfuric acid). An exceptionally large amount of water was adsorbed on MIL-100-F (>0.65 *g*_water_/*g*_adsorbent_) over the non-ionic functional MOF (0.5 *g*_water_/*g*_adsorbent_) and the adsorption amount remained stable even after 2000 adsorption/desorption cycles. Interestingly, the counter anion affects the water uptake, as it was observed that the values of *P/P_0_* for the steps are different. The steps in the isotherm of MIL-100-SO_4_ moved to lower *P/P_0_* values compared with those of the other compounds. In the meantime, the isosteric heats of adsorption (*q_st_*) on MIL-100-SO_4_ showed the largest values and MIL-100-Cl demonstrated the smallest values, a tendency similar to the hydration energies. According to the hydration energy and isosteric heat of adsorption, the interaction between water and a sulfate anion is stronger than that between water and a chloride anion. As a result, the adsorption for MIL-100-SO_4_ begins at a lower pressure than for MIL-100-Cl. Monte Carlo simulations in a grand canonical ensemble (GCMC) were carried out to confirm the influence of the three anions in the MIL-100(Fe) structures (e.g., F−, Cl−, or OH−) for water adsorption isotherms [83]. In the simulations, the water adsorption behavior and the relative structural stability of MIL-100(Fe) were investigated. The small cages, which provide a stronger interaction with water molecules compared to the large cages, were completely filled with water at a notably lower pressure. Among the three structures, because of the strongest interaction between the terminal F^-^ anion and water molecules, the adsorption isotherm of MIL-100(Fe)−F displayed the highest adsorption capability with the occurrence of water condensation at a much smaller relative pressure. It can be concluded that the counter anions incorporated in the structure tune the guest uptake, although adsorption properties of MOFs are usually changed by the pore size or organic ligands of the frameworks.

**Figure 11:**
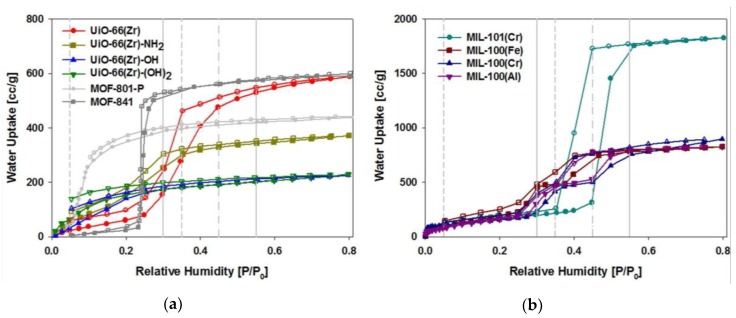
Water adsorption/desorption isotherms of the series of MOFs at 298 K. Reproduced with permission from [84]. Elsevier, 2016.

A series of isostructural UiO-66(Zr) with different functional groups, such as -NH_2_, -OH, and -(OH)_2_, were employed to functionalize the BDC linker to investigate the hydrophilic effect of the groups [84]. All the functionalized UiO-66(Zr) materials showed enhanced water adsorption at a low *P/P_0_* relative pressure due to the augmented interactions with water molecules, leading to isotherms that approached the type I shape (Figure 11a). However, the water adsorption of the functionalized UiO-66(Zr) materials in the high *P/P_0_* decreased significantly compared with the original UiO-66(Zr) as a consequence of the decreased surface area due to the insertion of bulky groups in the pores. The hysteresis in water adsorption and desorption isotherms, especially under low relative humidity conditions, may be elucidated by rehydroxylation of Zr-clusters during the water adsorption [52,85]. On the other hand, the unsaturated metal sites influenced the water adsorption/desorption and a study on the metal series of isostructural MIL-100(M) materials (M = Cr, Fe, and Al) was performed. As displayed in Figure 11b, the water adsorption isotherms of MIL-100(Cr), MIL-100(Fe), and MIL-100(Al) almost coincided with one another. The isotherm types were similar to type IV, but two steps at which the water loadings noticeably increased were clearly observed at *P/P_0_* = 0.25 and 0.4 for all three MOFs. These two steps revealed that capillary condensations occur step-by-step in two types of pores. The water uptake of the three MIL-100(M) materials at *P/P_0_* = 0.8 was approximately 820–900 cm^3^/g, which is approximately 45–50% compared with MIL-101(Cr). The water uptakes of MIL-101(Cr) and MIL-100(M) at *P/P_0_* = 0.8 correlated well with the surface areas (Brunauer–Emmett–Teller, BET). These results indicate that the type of unsaturated metal in MIL-100(M) does not significantly affect the water adsorption. Nonetheless, some interesting cyclic water adsorption/desorption behaviors depend on the type of unsaturated metal.

### 4.2. Functionalized MOFs with Composite Materials

Composite adsorbents are studied and developed with mainly two goals: (1) to improve the heat and mass transfer performance of chemical adsorbents, especially via the swelling, and (2) to increase the adsorption quantity of physical adsorbents via agglomeration phenomena. The composite adsorbents made from porous materials and chemical sorbents are commonly a combination.

**Figure 12:**
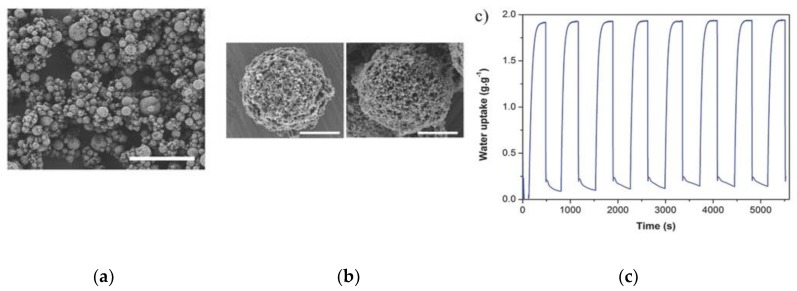
(**a**) Representative FESEM image of microspherical CaCl_2_@UiO-66, (**b**) FESEM images of microspherical CaCl_2_@UiO-66_38 superstructures before (left) and after (right) incubation in ethanol, (**c**) adsorption and desorption cycles for CaCl_2_@UiO-66_38. Scale bars: (**a**) 20 μm, (**b**) 3 μm. Reproduced with permission from [89]. Wiley, 2016.

Encapsulation of inorganic compounds in porous materials was explored to enhance the water uptake. The porous materials act as a media to disperse the salt particles and can provide good heat and mass transport for these salt particles. To date, different porous materials have been explored for encapsulation, including silica, filosilicates, activated carbon, and microporous zeolites [86,87,88]. Recently, the spherical hollow superstructure/beads of UiO-66 and UiO-66-NH_2_ encapsulated with inorganic salts were developed via spray drying. The inorganic salts CaCl_2_ and LiCl were selected for encapsulation during the assembly of the nanosized crystals [89]. The water sorption isotherm of CaCl_2_@UiO-66 at 298 K showed two segments with a steep increase in the water uptake (Figure 12). These two steps were attributed to the formation of CaCl_2_⋅0.33H_2_O at a relative humidity (RH) of 3% (water uptake of 0.15 *g*_water_/*g*_adsorbent_) and to the further transformation of this hydrate to CaCl_2_⋅2H_2_O at RHs ranging from 10% to 16% (water uptake of 0.33 *g*_water_/*g*_adsorbent_). Thereafter, the sorption curve ascended monotonically, indicating the formation of an aqueous solution of the salt and reaching a maximum water uptake of 1.93 *g*_water_/*g*_adsorbent_ at an RH of 90% [90,91]. Interestingly, a hysteresis loop at low pressures (*P*/*P_0_* = 0.10–0.16) was observed in the desorption branch due to the structural changes in the transition from CaCl_2_⋅2H_2_O hydrate to CaCl_2_⋅0.33H_2_O hydration. A hypothetical mechanism for the water sorption was suggested and the following steps take place: the anhydrous CaCl_2_ particles confined in the micropores of UiO-66 and/or in the inter-particular voids of the superstructures adsorb water and transform into crystalline CaCl_2_⋅0.33H_2_O. Then, this hydrate adsorbs more water and is converted to crystalline CaCl_2_⋅2H_2_O, and finally the salt is completely dissolved filling the pores and/or voids. Remarkably, the maximum uptake at 90% RH (1.93 *g*_water_/*g*_adsorbent_) remained constant with the number of cycles, confirming the stability of this composite to water sorption/desorption processes (Figure 2c). On the other hand, CaCl_2_@UiO-66-NH_2__38 showed a maximum uptake of 1.76 *g*_water_/*g*_adsorbent_, which is lower compared with its analogue based on UiO-66, but it retained 0.12 *g*_water_/*g*_adsorbent_ (6.8% of the total uptake) of water at an RH of 0%, which is double the amount of that of its UiO-66 analogue.

**Figure 13:**
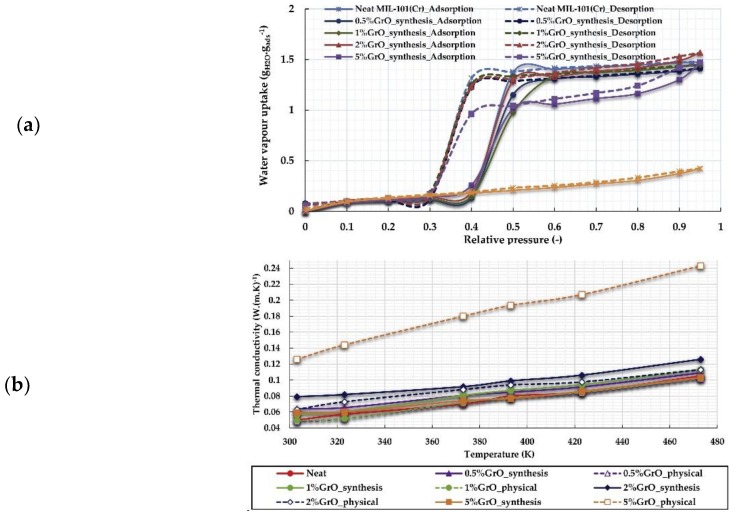
(**a**) Water adsorption isotherms and (**b**) Thermal conductivity of MIL-101(Cr) and Matérial Institute Lavoisier (MIL)/graphene oxide (GO) composites. Reproduced with permission from [96]. Elsevier, 2017.

Another route to improve the adsorption performance of MOFs can be realized via increasing the atomic density of the adsorbent material. For example, MOFs combined with dense carbon substrates, e.g., graphene oxide (GO), can produce composites (MOF@GO) with excellent adsorption performances, such as MOF-5@GO [92], MIL-101(Cr)@GO [93], and Cu-BTC@GO [94]. It was reported that the incorporation of GO into MOFs structures would noticeably improve the adsorption performances of the composites for adsorption of CO_2_, H_2_O, and volatile organic compounds (VOCs). A series of composites Cu-BTC@GO with various GO loadings was synthesized at room temperature [95]. The Cu-BTC@GO composite demonstrated a superhigh adsorption capacity for ethanol up to 13.60 mmol/g at 303 K, which was attributed to the introduction of GO leading to an increase in the surface dispersive forces and the mesoporous volume of Cu-BTC@GO. The ethanol adsorption capacity was higher than many other MOFs, including Cu-BTC, MIL-101, and MIL-53, under similar conditions. The MOF composite’s exceptional adsorption properties paid the price via extremely low thermal conductivity due to their large pore sizes and high free volumes. The low thermal conductivity limits the ability of the heat transfer processes to reach the desired operating temperatures quickly during both the adsorption and desorption phases. The thermal properties in addition to the hydrophilicity (as the oxygen in GO strongly binds to water molecules through hydrogen-bonding interactions) of the GO was the motivation to investigate the characteristics of MIL-101(Cr)/GO composites regarding water adsorption and thermal conductivity [96]. Two synthesized composites (with 2 wt% GO and 5 wt% GO) showed an increased uptake in the high relative pressure range, which is consistent with the suggestion that the oxygen functionalities of GO (hydroxyl, carboxyl, and epoxy) were able to coordinate to the metallic centers of the MIL-101(Cr) structure and hence new pores were created at the interface of the structure and the graphene layers. The 2 wt% GO composite showed a maximum water uptake of 1.56 *g*_water_/*g*_adsorbent_ while the 5 wt% GO composite reached a value of 1.47 g_water_/g_adsorbent_. The small reduction in water adsorption for the 5 wt% GO composite may be attributed to the fact that GO is a non-porous material and may cause pore blocking and hence lower the accessible surface area and pore volume of the composites if more GO is incorporated into MIL-101(Cr) (Figure 13). Based on the equilibrium data, the water loading difference, which is the difference between the amount of water adsorbed in the adsorption process and the amount of water remaining in the pores after the desorption process, is 1.36 *g*_water_/*g*_adsorbent_ for the parent material while it is 1.33 *g*_water_/*g*_adsorbent_ for the composite. Those values are much higher than those reported for other types of MOFs under similar operating conditions. Aluminum fumarate can have a water-loading difference of 0.45 *g*_water_/*g*_adsorbent_ [56], while MIL-100(Al) reaches a value of 0.4 *g*_water_/*g*_adsorbent_ and MIL-100(Fe) reaches 0.65 *g*_water_/*g*_adsorbent_ [43]. Furthermore, good thermal conductivity is crucial in adsorption and desorption processes. Figure 13 shows the thermal conductivity of the material as a function of temperature. It can be seen that the thermal conductivity of neat MIL-101(Cr) is 0.05 W (m K)^–1^ at 303 K and increases linearly to reach 0.103 W (m K)^–1^ at 473 K. The effect of the presence of graphene oxide at different ratios was also investigated. Despite the presence of the oxygen atoms, which typically enhances phonon scattering, the enhancement of the thermal conductivity observed could be attributed to the increase in the interlayer coupling due to interactions between the oxygen atom of the GO and the MIL-101(Cr) framework [97].

Because MOF materials can have a rather high surface areas, this topic merits attention. Another advantage of these MOFs is the relatively low temperatures needed during the desorbing of the working fluid (water) since this is mainly physical adsorption. However, it is also essential to check cautiously their properties, especially their hydrothermal stabilities. Observations obtained from several MOFs indicated that they are not stable in operational conditions, such as the presence of water and/or at relatively high temperatures, which results in dramatic decreases in the adsorption capacities and/or structure decomposition. The hydrothermal stability of MOFs can also be improved by shielding the metal–linker bond from water vapor using sterically demanding and hydrophobic linkers. However, as in many cases an aqua ligand or a free coordination site on the metal atom serves as an anchor for water cluster formation, hydrophilicity is usually strongly reduced in such cases, and the MOF may not adsorb any water at all [41]. For example, zinc imidazolate, ZIF-8, is hydrothermally stable because no water is adsorbed [98,99]. A zinc-based MOF showed increased hydrothermal stability when water adsorption is prevented due to interpenetration or pore blocking [100]. For use in adsorption heat transformation, hydrothermal stability cannot be deduced merely by retrieving the MOF from an aqueous suspension without structural damage but needs to be verified through a larger number of water vapor adsorption/desorption cycles. It still seems to be a challenge to find materials which have relatively high water uptake in a narrow relative humidity range while maintaining high structural integrity. The attempts to increase the water stability of MOFs have led to declines in their water uptake capacity due to enhanced hydrophobicity and reduction in surface areas.

## 5. Outlook

Metal–Organic Frameworks (MOFs) are new porous materials having a high surface area, pore size, and volume of which the geometry and properties can be tuned for the required application. Moreover, a high capability of solid–gas adsorption is a conversional criterion for a high potential as an adsorbent for heat transformation applications. However, it is not a general rule that a strong “adsorbed–adsorbent” interaction is obviously favorable to obtain a high adsorption capacity of the adsorbed vapor (working fluid) since a strong interaction requires a too-high desorption temperature (>473–573 K), which might not be provided from common heat sources (solar radiation, engine wastes, etc.). Thus, the high adsorption capacity caused by a strong adsorption interaction becomes, to the contrary, unfavorable for the transformation of low-temperature heat. On the other hand, a too-weak “adsorbed–adsorbent” interaction is auspicious for desorption; however, it does not allow during the adsorption phase sufficient driving force for adsorbed “sucking” and, hence, for cold generation. Thus, an optimal adsorbent should provide a moderate affinity toward adsorbed molecules that depends on particular boundary conditions of the cycle.

MOFs normally occur after synthesis as a fine powder and they are not applicable in most industrial processes, nor for heat transformation. The integration of MOFs as adsorbent and heat exchanger are new efficient applications and can be applied under real operating conditions of heat transformation technologies. Considering the thermodynamics, the former configuration (Heat transformation systems) is very simple and ensures good vapor transport but is considered to suffer from poor heat transfer due to the high thermal resistance between the adsorbent grains. As a result, the adsorption kinetics is expected to be slow and limited by the heat transfer. Coated MOF adsorbent material, in which the MOFs are directly connected to the surface (fins tube exchanger) or with a binder (ex situ) could directly solve the former issue. Elimination of the limitations originating from inefficient heat transfer inside the adsorber as well as the easy manipulation of the adsorbent thickness, in a manner avoiding mass transfer limitations, may be achieved by using coatings. Significant improvements have been demonstrated to take place in these systems when the adsorbent is directly crystallized on metal heat exchanger surfaces as coatings instead of being used in powder or pellet form [101]. The approach in which a binder is applied to produce a coating is based on a procedure developed previously for zeolites and direct crystallization via a thermal gradient method. In situ coatings are now the most popular method for heat transformation applications [102,103,104]. As mentioned before, MOF coatings can generally be produced via two routes: Binder-based coatings and direct crystallization. Still, several key factors in the design and manufacturing, such as layer thickness and the binder type, influence the heat transfer coefficient, the mechanical stability of such coatings, the coating texture, etc., and need further improvement.

Macrocellular foam composite adsorbent material is a new approach for an innovative adsorber, and is not necessarily an alternative to the adsorbent coating process [105,106]. The principle is based on the use of composite-adsorbent-filled silicone foams that can improve the adsorption surface area without altering the adsorption pump dynamic performances. Practical problems arising from an adsorbent is that the high thickness bed enhances the mass transfer and thermal conductivity. A directly coated adsorbent on the heat exchanger wall (Fins) displays a limited heat transfer between the heat exchanger surface and the adsorbent such that the mass transfer resistance is substantially reduced due to the very thin adsorbent layer [103,107]. However, multiple depositions are necessary in order to reach an acceptable adsorbent layer thickness (<0.1 mm). Moreover, in this approach, the adsorbent material can be homogeneously distributed along the surface of macrocellular foam generating a product with significant advantages in terms of the cheapness of the synthesis process, a low weight and large adsorbent thickness, high vapor diffusion permeability, and good hydrothermal and mechanical stability. The zeolite-embedded silicon foam evidenced the effective sorption and desorption processes, giving to composite adsorbents materials with silicon foam greater advantages than other zeolite-containing systems (e.g. coating, picking, direct growth, etc.) for heat pump applications [101].

## 6. Conclusions 

The adsorbent material is a key factor in the design and manufacturing of this application. In principle, a huge number of MOFs have already been synthesized, but the adsorption heat transformation has been studied for only a few of them,. We believe that future screening of MOFs as adsorbent materials for heat transformation applications will reveal novel and highly efficient working adsorbents. The development of novel adsorbents is needed in order to propel adsorption-based water adsorption from scientific curiosities into real-life applications. Next-generation adsorbents should have: (i) a high chemical stability to water, (ii) a tailorable hydrophilicity, and (iii) an adjustable pore diameter to fine-tune the adsorption profile and modulate the sorption kinetics. However, consideration to develop this technology has until now been more focused on the decrease of unit costs and the increase in energy efficiency. Adsorption is focused on finding more efficient working pairs, and for storage, the first prototypes are tested and new ones are designed with different or enhanced storage materials and new reactor concepts to optimize the energy output. All in all, we hope that this review will give new impulses to target-oriented research on novel adsorbents based on MOFs and their derivative modifications, which may be beneficial for further heat transformation applications.

## Figures and Tables

**Table 1:** Summary of potential MOFs and their properties in water adsorption. Reproduced with permission from [32], Tomsk Polytechnic University, 2018.

MOFs	Metals	Linkers	Surface area (m^2^·g^–1^)	Pore diameter (nm)	Pore volume (cm^3^·g^–1^)	Uptake* (cm^3^·g^–1^)	references
CAU-10	Al	1,3-H_2_BDC	635	0.7	0.25	0.31	[39]
CAU-10-H	Al	1,3-H_2_BDC	635	n.d.	0.5	0.382	[39]
CAU-10-NH_2_	Al	5-H_2_BDC-NH_2_	n.d.	n.d.	n.d.	0.19	[39]
CAU-10-NO_2_	Al	5-H_2_BDC-NO_2_	440	n.d.	0.18	0.15	[39]
CAU-10-OCH_3_	Al	5-methoxyiso-phthalic acid	n.d.	n.d.	n.d.	0.07	[39]
CAU-10-OH	Al	5- H_2_BDC-OH	n.d.	n.d.	n.d.	0.27	[39]
CAU-6	Al	BDC- NH_2_	620	n.d.	0.25	0.485	[40]
DUT-4	Al	H_2_NDC	1360	n.d.	0.79	0.28	[41]
DUT-67	Zr	H_2_TDC	1560	1.66/0.88	0.60	0.625	[29]
MIL-100	Cr	H_3_BTC	1517	2.5/2.9	n.d.	0.41	[42]
MIL-100	Fe	H_2_BDC	1549	n.d.	0.82	0.81	[41]
			1917	2.5/2.9	1.0	0.77	[43]
MIL-100	Al	H_3_BTC	1814	2.5/2.9	1.14	0.50	[43]
MIL-100	Cr	H_3_BTC	1330	2.5/2.9	0.77	0.40	[44]
			2059	2.9/3.4	1.103	1.01	[45]
			3017	n.d.	1.61	1.28	[41]
			3124	2.9/3.4	1.58	1.40	[46]
MIL-100-DEG	Cr	H_3_BTC	580	1.2/1.5/1.9	0.50	0.33	[44]
MIL-100-EG	Cr	H_3_BTC	710	1.2/1.5/1.9	0.47	0.43	[44]
MIL-101-NH_2_	Cr	H_2_BDC	2509	<2.9/3.4	1.27	0.90	[46]
			2690	<2.9/3.4	1.60	1.06	[47]
MIL-101-NO_2_	Cr	H_2_BDC	2146	<2.9/3.4	1.19	1.08	[46]
			1245	<2.9/3.4	0.7	0.44	[47]
MIL-101-*p*NH_2_	Cr	H_2_BDC	2495	<2.9/3.4	1.44	1.05	[47]
MIL-101-*p*NO_2_	Cr	H_2_BDC	2195	<2.9/3.4	1.11	0.6	[47]
MIL-101-soc	Cr	H_4_TCPT	4549	n.d.	2.1	1.95	[38]
MIL-125	Ti	H_2_BDC	1160	0.6/1.1	0.47	0.36	[30]
MIL-125-NH_2_	Ti	H_2_BDC-NH_2_	830	0.6/1.1	0.35	0.36	[30]
			1220	0.6/1.26	0.55	0.37	[48]
MIL-53	Al	H_2_BDC	1040	0.7-1.3	0.51	0.09	[30]
			n.d.	n.d.	n.d.	0.09	[49]
MIL-53-NH_2_	Al	H_2_BDC-NH_2_	940	0.7-1.3	0.37	0.05	[30]
			n.d.	n.d.	n.d.	0.09	[49]
MIL-53-OH	Al	H_2_BDC-OH	n.d.	n.d.	n.d.	0.40	[49]
MIL-53	Ga	H_2_BDC	1230	0.8-2	0.47	0.05	[30]
MIL-53-NH_2_	Ga	H_2_BDC-NH_2_	210	0.8-2	n.d.	0.02	[30]
MIL-53-(COOH)_2_	Fe	H_2_BDC-(COOH)_2_	n.d.	n.d.	n.d.	0.16	[49]
MIL-68	In	H_2_BDC	1100	0.6/1.6	0.42	0.32	[30]
MIL-68-NH_2_	In	H_2_BDC-NH_2_	850	0.6/1.6	0.302	0.32	[30]
MOF(NDI-SEt)	Zn	Pyrazole ligands	888	n.d.	1.6	0.25	[50]
MOF(NDI-SO_2_Et)	Zn	Pyrazole ligands	764	n.d.	<1.6	0.25	[50]
MOF(NDI-SOEt)	Zn	Pyrazole ligands	927	n.d.	<1.6	0.30	[50]
MOF-199	Cu	H_3_BTC	1340	n.d.	0.72	0.55	[41]
			921	2.1	0.492	0.64	[51]
			1270	0.9,0.6	0.62	0.49	[52]
MOF-74	Co	DOT	1130	1.11	0.49	0.63	[29]
MOF-74	Mg	DOT	1250	1.11	0.53	0.75	[29]
			1400	1.1	0.65	0.62	[52]
MOF-74	Ni	DOT	1040	1.11	0.46	0.615	[29]
			639	2.3	0.362	0.48	[51]
MOF-801-P	Zr	Fumaric acid	990	0.74,0.56,0.48	0.45	0.450	[29]
MOF-801-SC	Zr	Fumaric acid	690	0.74,0.56,0.48	0.27	0.35	[29]
MOF-802	Zr	PZDC	1290	0.84,0.74	0.49	0.11	[29]
MOF-804	Zr	DOT	1145	0.72,0.68	0.46	0.29	[29]
MOF-805	Zr	NDC-(OH)_2_	1230	0.95,0.86	0.48	0.415	[29]
MOF-806	Zr	BPDC-(OH)_2_	2220	1.26,1.01	0.85	0.425	[29]
MOF-808	Zr	BTC	2060	1.84	0.84	0.735	[29]
MOF-841	Zr	H_4_MTB	1390	0.92	0.53	0.640	[29]
PIZOF-2	Zr	PEDB-(OMe)_2_	2080	1.76	0.88	0.850	[29]
SIM-1	Zn	4-methyl-5-imidazolecarboxaldehyde	570	0.65	0.303	0.14	[30]
UiO-66	Zr	H_2_BDC	1290	0.84,0.74	0.49	0.535	[29]
			1032	0.75/1.2	0.77	0.40	[48]
			1105		0.55	0.39	[53]
			1160	0.6	0.52	0.37	[52]
UiO-66-1,4-Naphyl	Zr	1,4-Naphyl	757	n.d.	0.42	0.26	[53]
UiO-66-2,5-(OMe)_2_	Zr	2,5-(OMe)_2_	868	n.d.	0.38	0.42	[53]
UiO-66-NH_2_	Zr	H_2_BDC-NH_2_	1328	0.75/1.2	0.70	0.38	[48]
			1123	<0.75/1.2	0.52	0.34	[53]
			1040	0.6	0.57	0.37	[52]
UiO-66-NO_2_	Zr	H_2_BDC-NO_2_	792	<0.75/1.2	0.40	0.37	[53]
UiO-67	Zr	H_2_BPDC	2064	1.2/1.6	0.97	0.18	[48]
ZIF-8	Zn	2-MIM	1255	n.d.	0.64	0.02	[41]
			1530	1.1	0.485	0.01	[30]

*Water adsorption properties measured at 298 K at nearly saturated vapor pressure (*P/P_0_* ≈ 1), n.d. = no data. Ligand abbreviation: 1,3-H_2_BDC = 1,3-benzenedicarboxylic acid/1,4-Naphyl = 1,4-naphthalenedicarboxylic acid/2,5-(OMe)_2_ = 2,5-dimethoxy-terephthalic acid/2-MIM = 2-methylimidazole/5-H_2_BDC-NH_2_ = 5-aminoisophthalic acid/5-H_2_BDC-NO_2_ = 5-nitroisophthalic acid/5-H_2_BDC-OH = 5-hydroxyisophthalic acid/DEG = diethylene glycol/DOT or H_2_BDC-(OH)_2_= 2,5-dihydroxy-1,4-benzenedicarboxylic acid/EN = ethylenediamine/H_2_BDC = 1,4-benzenedicarboxylic acid/H_2_BDC-(COOH)_2_ = 1,2,4,5-benzenetetracarboxylic acid/H_2_BDC-NH_2_= 2-aminoterephthalic acid/H_2_BDC-NO_2_= 2-nitro-terephthalic acid/H_2_BDC-OH = 2-hydroxyterephthalic acid/H_2_BPDC = biphenyl-4,4´-dicarboxylic acid/H_2_BPDC-(OH)_2_= 3,3'-dihydroxy-4,4'-biphenyldicarboxylic acid / H_2_NDC-(OH)_2_ =1,5-dihydroxynaphthalene-2,6-dicarboxylic acid/H_2_-PEDB-(OMe)_2_= 4,4'-[(2,5-dimethoxy-1,4-phenylene)bis(ethyne-2,1-diyl)]dibenzoic acid/H_2_PZDC = 1H-pyrazole-3,5-dicarboxylic acid/H_2_TDC = thiophene-2,5-dicarboxylic acid/H_3_BTC = 1,3,5-benzenetricarboxylic acid/H_4_MTB = 4,4',4'',4'''-methanetetrayltetrabenzoicacid/H_4_TCPT = 3,3’’,5,5’’-tetrakis(4-carboxyphenyl)-p-terphenyl.

## References

[1] Isaac M., Van Vuuren D.P. (2009). Modeling global residential sector energy demand for heating and air conditioning in the context of climate change. Energy Policy.

[2] Alefeld G., Radermacher R. (1993). Heat Conversion Systems.

[3] Schmidt F., Henninger S., Stach H., Jänchen J., Henning H. Novel adsorbents for solar cooling applications. Proceedings of the International Conference Solar Air Conditioning.

[4] Henning H.-M. (2007). Solar assisted air conditioning of buildings–an overview. Appl. Therm. Eng..

[5] Demir H., Mobedi M., Ülkü S. (2008). A review on adsorption heat pump: Problems and solutions. Renew. Sustain. Energy Rev..

[6] Ng K., Chua H., Chung C., Loke C., Kashiwagi T., Akisawa A., Saha B. (2001). Experimental investigation of the silica gel–water adsorption isotherm characteristics. Appl. Therm. Eng..

[7] Furukawa H., Cordova K.E., O’Keeffe M., Yaghi O.M. (2013). The Chemistry and Applications of Metal-Organic Frameworks. Science.

[8] He Y., Zhou W., Qian G., Chen B. (2014). Methane storage in metal–organic frameworks. Chem. Soc. Rev..

[9] Adil K., Belmabkhout Y., Pillai R.S., Cadiau A., Bhatt P.M., Assen A.H., Maurin G., Eddaoudi M. (2017). Gas/vapour separation using ultra-microporous metal–organic frameworks: Insights into the structure/separation relationship. Chem. Soc. Rev..

[10] Rogge S.M.J., Bavykina A., Hajek J., Garcia H., Olivos-Suarez A.I., Sepúlveda-Escribano A., Vimont A., Clet G., Bazin P., Kapteijn F. (2017). Metal–organic and covalent organic frameworks as single-site catalysts. Chem. Soc. Rev..

[11] Wuttke S., Lismont M., Escudero A., Rungtaweevoranit B., Parak W.J. (2017). Positioning metal-organic framework nanoparticles within the context of drug delivery—A comparison with mesoporous silica nanoparticles and dendrimers. Biomater.

[12] Freund R., Lächelt U., Gruber T., Rühle B., Wuttke S. (2018). Multifunctional Efficiency: Extending the Concept of Atom Economy to Functional Nanomaterials. ACS Nano.

[13] Lismont M., Dreesen L., Wuttke S. (2017). Metal-Organic Framework Nanoparticles in Photodynamic Therapy: Current Status and Perspectives. Adv. Funct. Mater..

[14] James S.L. (2003). Metal-organic frameworks. Chem. Soc. Rev..

[15] Chaemchuen S., Kabir N.A., Zhou K., Verpoort F. (2013). Metal–organic frameworks for upgrading biogas via CO 2 adsorption to biogas green energy. Chem. Soc. Rev..

[16] Chughtai A.H., Ahmad N., Younus H.A., Laypkov A., Verpoort F. (2015). Metal–organic frameworks: Versatile heterogeneous catalysts for efficient catalytic organic transformations. Chem. Soc. Rev..

[17] Hendon C.H., Rieth A.J., Korzyński M.D., Dincă M. (2017). Grand Challenges and Future Opportunities for Metal–Organic Frameworks. ACS Cent. Sci..

[18] Henninger S.K., Jeremias F., Kummer H., Janiak C. (2012). MOFs for use in adsorption heat pump processes. Eur. J. Inorg. Chem..

[19] Younos T., Tulou K.E. (2005). Overview of desalination techniques. J. Contemp. Phys..

[20] Youssef P.G., Dakkama H., Mahmoud S.M., AL-Dadah R.K. (2017). Experimental investigation of adsorption water desalination/cooling system using CPO-27Ni MOF. Desalination.

[21] Wang X., Ng K.C. (2005). Experimental investigation of an adsorption desalination plant using low-temperature waste heat. Appl. Therm. Eng..

[22] Ng K.C., Xiao-Lin W., Gao L., Chakraborty A., Saha B.B., Koyama S., Akisawa A., Kashiwagi T. (2013). Apparatus and Method for Desalination. US patent.

[23] Thu K., Ng K.C., Saha B.B., Chakraborty A., Koyama S. (2009). Operational strategy of adsorption desalination systems. Int. J. Heat Mass Transfer.

[24] Henninger S., Schmidt F., Henning H.-M. (2010). Water adsorption characteristics of novel materials for heat transformation applications. Appl. Therm. Eng..

[25] Brancato V., Frazzica A. (2018). Characterisation and comparative analysis of zeotype water adsorbents for heat transformation applications. Sol. Energy Mater. Sol. Cells.

[26] You W., Liu Y., Howe J.D., Sholl D.S. (2018). Competitive Binding of Ethylene, Water, and Carbon Monoxide in Metal–Organic Framework Materials with Open Cu Sites. J. Phys. Chem. C.

[27] Tatlier M., Munz G., Henninger S.K. (2018). Relation of water adsorption capacities of zeolites with their structural properties. Microporous Mesoporous Mater..

[28] Aristov Y.I. (2007). Novel materials for adsorptive heat pumping and storage: Screening and nanotailoring of sorption properties. J. Chem. Eng. Jpn..

[29] Furukawa H., Gándara F., Zhang Y.-B., Jiang J., Queen W.L., Hudson M.R., Yaghi O.M. (2014). Water Adsorption in Porous Metal–Organic Frameworks and Related Materials. J. Am. Chem. Soc..

[30] Canivet J., Bonnefoy J., Daniel C., Legrand A., Coasne B., Farrusseng D. (2014). Structure–property relationships of water adsorption in metal–organic frameworks. New J. Chem..

[31] Burtch N.C., Jasuja H., Walton K.S. (2014). Water stability and adsorption in metal–organic frameworks. Chem. Rev..

[32] Chaemchuen S., Wang J.C., Gilani A.G., Verpoort F. (2018). Metal-organic frameworks applied for water purification. Resour. Efficient Technol..

[33] Taylor J.M., Vaidhyanathan R., Iremonger S.S., Shimizu G.K. (2012). Enhancing water stability of metal–organic frameworks via phosphonate monoester linkers. J. Am. Chem. Soc..

[34] Canivet J., Fateeva A., Guo Y., Coasne B., Farrusseng D. (2014). Water adsorption in MOFs: Fundamentals and applications. Chem. Soc. Rev..

[35] Jasuja H., Burtch N.C., Huang Y.-G., Cai Y., Walton K.S. (2013). Kinetic water stability of an isostructural family of zinc-based pillared metal–organic frameworks. Langmuir.

[36] Li S., Chen Y., Pei X., Zhang S., Feng X., Zhou J., Wang B. (2016). Water Purification: Adsorption over Metal-Organic Frameworks. Chin. J. Chem..

[37] Li N., Xu J., Feng R., Hu T.-L., Bu X.-H. (2016). Governing metal–organic frameworks towards high stability. Chem. Commun..

[38] Towsif Abtab S.M., Alezi D., Bhatt P.M., Shkurenko A., Belmabkhout Y., Aggarwal H., Weseliński Ł.J., Alsadun N., Samin U., Hedhili M.N. (2018). Reticular Chemistry in Action: A Hydrolytically Stable MOF Capturing Twice Its Weight in Adsorbed Water. CHEM.

[39] Reinsch H., van der Veen M.A., Gil B., Marszalek B., Verbiest T., De Vos D., Stock N. (2012). Structures, sorption characteristics, and nonlinear optical properties of a new series of highly stable aluminum MOFs. Chem. Mater..

[40] Reinsch H., Marszalek B., Wack J., Senker J., Gil B., Stock N. (2012). A new Al-MOF based on a unique column-shaped inorganic building unit exhibiting strongly hydrophilic sorption behaviour. Chem. Commun..

[41] Küsgens P., Rose M., Senkovska I., Fröde H., Henschel A., Siegle S., Kaskel S. (2009). Characterization of metal-organic frameworks by water adsorption. Microporous Mesoporous Mater..

[42] Akiyama G., Matsuda R., Kitagawa S. (2010). Highly porous and stable coordination polymers as water sorption materials. Chem. Lett..

[43] Jeremias F., Khutia A., Henninger S.K., Janiak C. (2012). MIL-100(Al, Fe) as water adsorbents for heat transformation purposes-a promising application. J. Mater. Chem..

[44] Wickenheisser M., Jeremias F., Henninger S.K., Janiak C. (2013). Grafting of hydrophilic ethylene glycols or ethylenediamine on coordinatively unsaturated metal sites in MIL-100(Cr) for improved water adsorption characteristics. Inorg. Chim. Acta.

[45] Ehrenmann J., Henninger S.K., Janiak C. (2011). Water Adsorption Characteristics of MIL-101 for Heat-Transformation Applications of MOFs. Eur. J. Inorg. Chem..

[46] Akiyama G., Matsuda R., Sato H., Hori A., Takata M., Kitagawa S. (2012). Effect of functional groups in MIL-101 on water sorption behavior. Microporous Mesoporous Mater..

[47] Khutia A., Rammelberg H.U., Schmidt T., Henninger S., Janiak C. (2013). Water Sorption Cycle Measurements on Functionalized MIL-101Cr for Heat Transformation Application. Chem. Mater..

[48] Jeremias F., Lozan V., Henninger S.K., Janiak C. (2013). Programming MOFs for water sorption: Amino-functionalized MIL-125 and UiO-66 for heat transformation and heat storage applications. Dalton Trans..

[49] Shigematsu A., Yamada T., Kitagawa H. (2011). Wide control of proton conductivity in porous coordination polymers. J. Am. Chem. Soc..

[50] Wade C.R., Corrales-Sanchez T., Narayan T.C., Dincă M. (2013). Postsynthetic tuning of hydrophilicity in pyrazolate MOFs to modulate water adsorption properties. Energy Environ. Sci..

[51] Liu J., Wang Y., Benin A.I., Jakubczak P., Willis R.R., LeVan M.D. (2010). CO_2_/H_2_O Adsorption Equilibrium and Rates on Metal−Organic Frameworks: HKUST-1 and Ni/DOBDC. Langmuir.

[52] Schoenecker P.M., Carson C.G., Jasuja H., Flemming C.J., Walton K.S. (2012). Effect of water adsorption on retention of structure and surface area of metal–organic frameworks. Ind. Eng. Chem. Res..

[53] Cmarik G.E., Kim M., Cohen S.M., Walton K.S. (2012). Tuning the Adsorption Properties of UiO-66 via Ligand Functionalization. Langmuir.

[54] Henninger S.K., Habib H.A., Janiak C. (2009). MOFs as adsorbents for low temperature heating and cooling applications. J. Am. Chem. Soc..

[55] Saha B.B., El-Sharkawy I.I., Miyazaki T., Koyama S., Henninger S.K., Herbst A., Janiak C. (2015). Ethanol adsorption onto metal organic framework: Theory and experiments. Energy.

[56] Jeremias F., Frohlich D., Janiak C., Henninger S.K. (2014). Advancement of sorption-based heat transformation by a metal coating of highly-stable, hydrophilic aluminium fumarate MOF. RSC Adv..

[57] Gordeeva L.G., Solovyeva M.V., Aristov Y.I. (2016). NH2-MIL-125 as a promising material for adsorptive heat transformation and storage. Energy.

[58] Aristov Y.I., Tokarev M.M., Sharonov V.E. (2008). Universal relation between the boundary temperatures of a basic cycle of sorption heat machines. Chem. Eng. Sci..

[59] Aristov Y.I. (2013). Challenging offers of material science for adsorption heat transformation: A review. Appl. Therm. Eng..

[60] Chui S.S.-Y., Lo S.M.-F., Charmant J.P., Orpen A.G., Williams I.D. (1999). A chemically functionalizable nanoporous material [Cu_3_(TMA)_2_(H2O)_3_]_n_. Science.

[61] Rowsell J.L., Yaghi O.M. (2006). Effects of functionalization, catenation, and variation of the metal oxide and organic linking units on the low-pressure hydrogen adsorption properties of metal—organic frameworks. J. Am. Chem. Soc..

[62] Schlichte K., Kratzke T., Kaskel S. (2004). Improved synthesis, thermal stability and catalytic properties of the metal-organic framework compound Cu_3_(BTC)_2_. Microporous Mesoporous Mater..

[63] Prestipino C., Regli L., Vitillo J., Bonino F., Damin A., Lamberti C., Zecchina A., Solari P., Kongshaug K., Bordiga S. (2006). Local structure of framework Cu (II) in HKUST-1 metallorganic framework: Spectroscopic characterization upon activation and interaction with adsorbates. Chem. Mater..

[64] Kitaura R., Fujimoto K., Noro S.I., Kondo M., Kitagawa S. (2002). A Pillared-Layer Coordination Polymer Network Displaying Hysteretic Sorption:[Cu_2_(pzdc)_2_(dpyg)]_n_ (pzdc=Pyrazine-2, 3-dicarboxylate; dpyg=1, 2-Di (4-pyridyl) glycol). Angew. Chem. Int. Ed..

[65] Férey G., Mellot-Draznieks C., Serre C., Millange F., Dutour J., Surblé S., Margiolaki I. (2005). A chromium terephthalate-based solid with unusually large pore volumes and surface area. Science.

[66] Baerlocher C., McCusker L.B., Olson D.H. (2007). Atlas of Zeolite Framework Types.

[67] Llewellyn P.L., Bourrelly S., Serre C., Vimont A., Daturi M., Hamon L., De Weireld G., Chang J.-S., Hong D.-Y., Kyu Hwang Y. (2008). High Uptakes of CO_2_ and CH_4_ in Mesoporous Metal—Organic Frameworks MIL-100 and MIL-101. Langmuir.

[68] Chowdhury P., Bikkina C., Gumma S. (2009). Gas adsorption properties of the chromium-based metal organic framework MIL-J. Phys. Chem. C.

[69] Elsayed E., Raya A.-D., Mahmoud S., Anderson P.A., Elsayed A., Youssef P.G. (2017). CPO-27 (Ni), aluminium fumarate and MIL-101 (Cr) MOF materials for adsorption water desalination. Desalination.

[70] Rieth A.J., Dincă M. (2018). Tricking Inert Metals into Water-Absorbing MOFs. Joule.

[71] Elsayed A., Al-Dadah R., Mahmoud S., Shi B., Youessef P., Elshaer A., Kaialy W. Characterisation of CPO-27Ni Metal Organic Framework Material for Water Adsorption. Proceedings of the Sustainable Thermal Energy management Network (SUSTEM).

[72] Elsayed E., Raya A.-D., Mahmoud S., Elsayed A., Anderson P.A. (2016). Aluminium fumarate and CPO-27 (Ni) MOFs: Characterization and thermodynamic analysis for adsorption heat pump applications. Appl. Therm. Eng..

[73] Alvarez E., Guillou N., Martineau C., Bueken B., Van de Voorde B., Le Guillouzer C., Fabry P., Nouar F., Taulelle F., De Vos D. (2015). The structure of the aluminum fumarate metal–organic framework AAngew. Chem. Int. Ed..

[74] Loiseau T., Serre C., Huguenard C., Fink G., Taulelle F., Henry M., Bataille T., Ferey G. (2004). A rationale for the large breathing of the porous aluminum terephthalate (mil-53) upon hydration. Chem. Eur. J..

[75] De Lange M., Zeng T., Vlugt T., Gascon J., Kapteijn F. (2015). Manufacture of dense CAU-10-H coatings for application in adsorption driven heat pumps: Optimization and characterization. Cryst. Eng. Commun..

[76] Fröhlich D., Henninger S.K., Janiak C. (2014). Multicycle water vapour stability of microporous breathing MOF aluminium isophthalate CAU-10-H. Dalton Trans..

[77] Fröhlich D., Pantatosaki E., Kolokathis P.D., Markey K., Reinsch H., Baumgartner M., van der Veen M.A., De Vos D.E., Stock N., Papadopoulos G.K. (2016). Water adsorption behaviour of CAU-10-H: A thorough investigation of its structure–property relationships. J. Mater. Chem. A.

[78] Hamon L., Serre C., Devic T., Loiseau T., Millange F., Férey G., Weireld G.D. (2009). Comparative study of hydrogen sulfide adsorption in the MIL-53 (Al, Cr, Fe), MIL-47 (V), MIL-100 (Cr), and MIL-101 (Cr) metal− organic frameworks at room temperature. J. Am. Chem. Soc..

[79] Férey G. (2008). Hybrid porous solids: Past, present, future. Chem. Soc. Rev..

[80] Horcajada P., Surblé S., Serre C., Hong D.-Y., Seo Y.-K., Chang J.-S., Greneche J.-M., Margiolaki I., Férey G. (2007). Synthesis and catalytic properties of MIL-100 (Fe), an iron (III) carboxylate with large pores. Chem. Commun..

[81] Jasuja H., Zang J., Sholl D.S., Walton K.S. (2012). Rational tuning of water vapor and CO_2_ adsorption in highly stable Zr-based MOFs. J. Phys. Chem. C.

[82] Kim S.-N., Kim J., Kim H.-Y., Cho H.-Y., Ahn W.-S. (2013). Adsorption/catalytic properties of MIL-125 and NH2-MIL-Catal. Today.

[83] Chen Y.-R., Liou K.-H., Kang D.-Y., Chen J.-J., Lin L.-C. (2018). Investigation of the Water Adsorption Properties and Structural Stability of MIL-100 (Fe) with Different Anions. Langmuir.

[84] Kim S.-I., Yoon T.-U., Kim M.-B., Lee S.-J., Hwang Y.K., Chang J.-S., Kim H.-J., Lee H.-N., Lee U.-H., Bae Y.-S. (2016). Metal–organic frameworks with high working capacities and cyclic hydrothermal stabilities for fresh water production. Chem. Eng. J..

[85] Wiersum A.D., Soubeyrand-Lenoir E., Yang Q., Moulin B., Guillerm V., Yahia M.B., Bourrelly S., Vimont A., Miller S., Vagner C. (2011). An Evaluation of UiO-66 for Gas-Based Applications. Chem. Asian J..

[86] Tashiro Y., Kubo M., Katsumi Y., Meguro T., Komeya K. (2004). Assessment of adsorption-desorption characteristics of adsorbents for adsorptive desiccant cooling system. Asian J. Mater. Sci..

[87] Meunier F. (2013). Adsorption heat powered heat pumps. Appl. Therm. Eng..

[88] Bonaccorsi L., Calabrese L., Freni A., Proverbio E., Restuccia G. (2013). Zeolites direct synthesis on heat exchangers for adsorption heat pumps. Appl. Therm. Eng..

[89] Garzón-Tovar L., Pérez-Carvajal J., Imaz I., Maspoch D. (2017). Composite Salt in Porous Metal-Organic Frameworks for Adsorption Heat Transformation. Adv. Funct. Mater..

[90] Yuan Y., Zhang H., Yang F., Zhang N., Cao X. (2016). Inorganic composite sorbents for water vapor sorption: A research progress. Renew. Sustain. Energy Rev..

[91] Glaznev I., Ponomarenko I., Kirik S., Aristov Y. (2011). Composites CaCl2/SBA-15 for adsorptive transformation of low temperature heat: Pore size effect. Int. J. Refrig..

[92] Petit C., Bandosz T.J. (2010). Enhanced Adsorption of Ammonia on Metal-Organic Framework/Graphite Oxide Composites: Analysis of Surface Interactions. Adv. Funct. Mater..

[93] Yan J., Yu Y., Ma C., Xiao J., Xia Q., Li Y., Li Z. (2015). Adsorption isotherms and kinetics of water vapor on novel adsorbents MIL-101 (Cr)@ GO with super-high capacity. Appl. Therm. Eng..

[94] Petit C., Burress J., Bandosz T.J. (2011). The synthesis and characterization of copper-based metal–organic framework/graphite oxide composites. Carbon.

[95] Yan J., Yu Y., Xiao J., Li Y., Li Z. (2016). Improved Ethanol Adsorption Capacity and Coefficient of Performance for Adsorption Chillers of Cu-BTC@ GO Composite Prepared by Rapid Room Temperature Synthesis. Ind. Eng. Chem. Res..

[96] Elsayed E., Wang H., Anderson P.A., Al-Dadah R., Mahmoud S., Navarro H., Ding Y., Bowen J. (2017). Development of MIL-101 (Cr)/GrO composites for adsorption heat pump applications. Microporous Mesoporous Mater..

[97] Mahanta N.K., Abramson A.R. Thermal conductivity of graphene and graphene oxide nanoplatelets. Proceedings of the 13th InterSociety Conference on Thermal and Thermomechanical Phenomena in Electronic Systems.

[98] Zhou K., Mousavi B., Luo Z., Phatanasri S., Chaemchuen S., Verpoort F. (2017). Characterization and properties of Zn/Co zeolitic imidazolate frameworks vs. ZIF-8 and ZIF-J. Mater. Chem. A.

[99] Zhang J.P., Zhu A.X., Lin R.B., Qi X.L., Chen X.M. (2011). Pore Surface Tailored SOD-Type Metal-Organic Zeolites. Adv. Mater..

[100] Birsa Čelič T., Mazaj M., Guillou N., Elkaïm E., El Roz M., Thibault-Starzyk F., Mali G., Rangus M., Čendakdak T., Kaučič V. (2013). Study of hydrothermal stability and water sorption characteristics of 3-dimensional Zn-based trimesate. J. Phys. Chem. C.

[101] Tatlıer M., Tantekin-Ersolmaz B., Erdem-Şenatalar A. (1999). A novel approach to enhance heat and mass transfer in adsorption heat pumps using the zeolite–water pair. Microporous Mesoporous Mater..

[102] Bauer J., Herrmann R., Mittelbach W., Schwieger W. (2009). Zeolite/aluminum composite adsorbents for application in adsorption refrigeration. Int. J. Energy Res..

[103] Bonaccorsi L., Freni A., Proverbio E., Restuccia G., Russo F. (2006). Zeolite coated copper foams for heat pumping applications. Microporous Mesoporous Mater..

[104] Geus E.R., van Bekkum H., Bakker W.J., Moulijn J.A. (1993). High-temperature stainless steel supported zeolite (MFI) membranes: Preparation, module construction, and permeation experiments. Microporous Mater..

[105] Calabrese L., Bonaccorsi L., Freni A., Proverbio E. (2017). Silicone composite foams for adsorption heat pump applications. Sustain. Mater.Technol..

[106] Calabrese L., Bonaccorsi L., Freni A., Proverbio E. (2017). Synthesis of SAPO-34 zeolite filled macrocellular foams for adsorption heat pump applications: A preliminary study. Appl. Therm. Eng..

[107] Guilleminot J., Choisier A., Chalfen J., Nicolas S., Reymoney J. (1993). Heat transfer intensification in fixed bed adsorbers. Heat Recover. Syst. CHP.

